# Genome Sequence of *Listeria monocytogenes* Strain HPB5415, Collected during a 2008 Listeriosis Outbreak in Canada

**DOI:** 10.1128/genomeA.00637-15

**Published:** 2015-06-11

**Authors:** Arthur W. Pightling, Franco Pagotto

**Affiliations:** Listeriosis Reference Service for Canada, Microbiology Research Division, Bureau of Microbial Hazards, Food Directorate, Health Products and Food Branch, Health Canada, Ottawa, Ontario, Canada

## Abstract

*Listeria monocytogenes* strain HPB5415—isolated from deli meat—was found in 2008 to have the same pulsed-field gel electrophoresis patterns as a clinical strain (08-5923). However, whether nucleotide differences (single nucleotide polymorphisms [SNPs]) exist between their genomes was not determined. We sequenced the *L. monocytogenes* strain HPB5415 genome and identified 52 SNPs relative to strain 08-5923.

## GENOME ANNOUNCEMENT

*Listeria monocytogenes* is a Gram-positive pathogenic bacterium that naturally inhabits plant, soil, and surface water environments (1) but may also be present in ready-to-eat foods. Consumption of foods contaminated with *L. monocytogenes* can cause a severe life-threatening illness called listeriosis (1, 2). Listeriosis may result in a variety of symptoms (central nervous system infections, bacteremia, and endocarditis), especially in immunocompromised or elderly adults, while neonatal infections may result in abortions or stillbirths (3). In 2008, a listeriosis outbreak caused by contaminated deli meats encompassed seven Canadian provinces (Alberta, British Columbia, Manitoba, New Brunswick, Ontario, Quebec, and Saskatchewan), resulting in 57 confirmed clinical cases and 22 deaths (http://www.health.gov.on.ca/en/public/publications/disease/docs/listeriosis_outbreak_epi_sum.pdf). In Ontario, two clinical serotype 1/2a isolates (08-5578 [syn., HPB5622] and 08-5923 [syn., HPB5628]) were identified with pulsed-field gel electrophoresis (08-5578: ApaI = LMAAI.0001 and AscI = LMACI.0040; 08-5923: ApaI = LMAAI.0001 and AscI = LMACI.0001) and later compared with whole-genome sequencing (4). Strain HPB5415 was isolated during the outbreak from a sealed package of deli meat. The strain HPB5415 pulsed-field pattern is identical to that of strain 08-5923. However, the genome was not sequenced. Here, we present the draft genome sequence of strain HPB5415 and compare it to that of strain 08-5923.

Sequence data were generated and assembled as previously described (5, 6). Briefly, we prepared a paired-end library with the Nextera XT DNA sample preparation kit (Illumina, San Diego, California) and generated sequence data on a MiSeq Benchtop sequencer (Illumina) for 500 cycles. The reads were assembled *de novo* into a high-quality draft genome with SPAdes v3.0.0 (7), using the BayesHammer error correction tool (8). The assembly resulted in 25 nonoverlapping contiguous sequences with a total length of 2,979,429 nucleotides, 37.88% G+C content, and 168.3-fold sequencing coverage. Gene predictions and annotations were performed at the National Center for Biotechnology Information (NCBI) with the Prokaryotic Genome Annotation Pipeline (9). In total, 2,915 coding sequences, 12 pseudogenes, 1 clustered regularly interspaced short palindromic repeat (CRISPR) array, 57 transfer RNAs, and 1 noncoding RNA were identified.

In order to identify differences between chromosome sequences, we aligned the strain HPB5415 short-read sequence data (submitted to the NCBI sequence read archive under accession no. SRR2000564) to the *Listeria monocytogenes* strain 08-5923 chromosome sequence obtained from NCBI (NC_013768.1) with SMALT (http://www.sanger.ac.uk/resources/software/smalt/). We then used FreeBayes (10) to identify 52 single-nucleotide polymorphisms and 10 indels relative to the strain 08-5923 genome sequence. We also compared multilocus sequence typing (11) profiles of strain 08-5923 (*abcZ*-5*, bglA*-6*,* cat-2*, dapE-*29*, dat*-5*, ldh*-3*,* and *lkhA*-1; sequence type 120) and strain HPB5415 (*abcZ*-57, *bglA*-6, cat-2, *dapE*-29, *dat*-5, *ldh*-3, and *lhkA*-1; sequence type 292) and found that all loci are identical except *abcZ*, which differs at a single nucleotide position (118; 08-5923 = A and HPB5415 = G). These data indicate that, although the pulsed-field patterns are identical, there are differences between the strain HPB5415 and 08-5923 chromosome sequences.

### Nucleotide sequence accession numbers.

This whole-genome shotgun project was deposited at DDBJ/EMBL/GenBank under the accession no. JOKV00000000. The version described in this paper is the first version, JOKV01000000.
